# Effect of Temperature and Humidity on the Water and Dioxygen Transport Properties of Polybutylene Succinate/Graphene Nanoplatelets Nanocomposite Films

**DOI:** 10.3390/membranes12070721

**Published:** 2022-07-20

**Authors:** Raphaël Cosquer, Sébastien Pruvost, Fabrice Gouanvé

**Affiliations:** Univ Lyon, CNRS, UMR 5223, Ingénierie des Matériaux Polymères, Université Claude Bernard Lyon 1, INSA Lyon, Université Jean Monnet, CEDEX, F-69621 Villeurbanne, France; raphael.cosquer@insa-lyon.fr (R.C.); sebastien.pruvost@insa-lyon.fr (S.P.)

**Keywords:** graphene nanoplatelets, polybutylene succinate, nanocomposites, activation energy, water sorption, water and dioxygen permeability

## Abstract

Nanocomposite films of polybutylene succinate (PBS)/graphene nanoplatelets (GnP) with a GnP content ranging from 0 to 1.35 wt.% were prepared by melt processing. The morphology of both the neat PBS and PBS/GnP nanocomposites were investigated and revealed no significant impact of GnP on the crystalline microstructure. Moisture sorption at 10 °C, 25 °C, and 40 °C were analyzed and modeled using the Guggenheim, Andersen, and De Boer (GAB) equation and Zimm-Lundberg theory, allowing for a phenomenological analysis at the molecular scale. An understanding of the transport sorption properties was proposed by the determination of the molar heat of sorption (Δ*H_s_*), and the activation energy of the diffusion (*E_d_*) of water in the matrix since both solubility and diffusion are thermo-activable properties. Both Δ*H_s_* and *E_d_* showed a good correlation with the water clustering theory at high water activity. Water and dioxygen permeabilities ($P_{H_{2}O}$ and $P_{O_{2}}$) were determined as a function of temperature and water activity. $P_{H_{2}O}$ and $P_{O_{2}}$ decreased with the addition of a small amount of GnP, regardless of the studied temperature. Moreover, the evolution of $P_{H_{2}O}$ as a function of water activity was driven by the solubility process, whereas at a given water activity, $P_{H_{2}O}$ was driven by the diffusion process. Activation energies of the permeability (*E_p_*) of water and dioxygen showed a dependency on the nature of the permeant molecule. Finally, from the Δ*H_s_*, *E_d_*, and *E_p_* obtained values, the reduction in water permeability with the addition of a low content of GnP was attributed mainly to a tortuosity effect without diffusive interfaces rather than a significant change in the transport property mechanism.

## 1. Introduction

Due to the rapid depletion of fossil fuels and the vast amount of non-biodegradable plastics in use, biodegradable polymer-based materials are an interesting alternative to the usual non-biodegradable polymer regarding global environmental problems [1]. One way to develop new biodegradable polymers consists of using renewable resources as the raw materials. In this context, in recent years, both academic and industrial researchers have focused their attention on the use of biodegradable materials. Due to its bio-based production ranging from 0% to 100% and its biodegradability, polybutylene succinate (PBS) has been widely studied over the past decades. PBS is a versatile semi-crystalline polymer that provides a wide range of workability. The relatively low melting temperature of PBS allows for its manufacture through a variety of processing methods including extrusion and injection molding. PBS has similar physical properties to polyethylene [1]. PBS has greater thermal properties, melt processability, and chemical resistance than other aliphatic polyesters [2], making it a promising polymer material for various applications such as food packaging. However, due to the presence of an ester group in its chemical structure, the hydrolysis of PBS generates chains scission, leading to low molecular weight polymers [3]. PBS has also been listed as being certified as compostable according to the Biodegradable Products Institute and is available in direct food contact grades [2,4].

However, gas and water barrier properties are missing and can be improved by blending PBS with other materials such as high aspect ratio nanofillers to satisfy the application specifications [5,6]. Carbon-based fillers with a high aspect ratio (graphite, graphene, graphene oxide, etc.) can be good candidates for the improvement in the barrier properties by introducing a tortuous pathway in the amorphous phase.

To target a possible application of PBS in food packaging, understanding the transport behavior of small molecules in this polymer material is fundamental. To do so, the water sorption, water permeability, and dioxygen permeability characterization are essential to extend the shelf-life of packaged foods. The transport properties of small molecules in polymer materials are usually temperature and relative humidity dependent, which can greatly influence the shelf-life of packaged food. Even if PBS is defined as a hydrophobic polymer because it absorbs 1.5% of water (g_w_.g^−1^_polymer_) for a water activity equal to 0.9 [7], water molecules can have detrimental effects on its barrier properties. Cosquer et al., showed an increase in the water permeability of PBS from 1900 Barrer to 2500 Barrer at 25 °C for a water activity range from 0.5 to 1 and Charlon et al., found a similar value, 2616 Barrer at a water activity of 1 [7,8]. No study on the influence of temperature on the transport properties of PBS was reported in the literature, but Siparsky et al., showed an increase in the water permeability from 2133 Barrer to 2666 Barrer for poly(L-lactide) (PLA) when the temperature increased from 20 to 50 °C in the anhydrous state [9].

Moreover, a fundamental understanding of the degradation kinetics and mechanism is required in order to predict the degradation of PBS in various environments. As previously mentioned, the degradation mechanism of PBS is the hydrolysis of the ester groups in the aliphatic chain [10]. Therefore, the water solubility and diffusivity coefficients provide information on the water molecule content available in the polymer matrix and also the rate of the water molecule diffusion that takes place at a given temperature and water activity for hydrolysis. The results could be used to predict the rate of PBS degradation.

Thus, the purpose of this paper is to describe the dependency of the apparent coefficients of permeability, diffusion, and solubility of dioxygen and water with the temperature and water activity in the neat PBS and PBS/GnP nanocomposites. We also tried to establish relationships between the structural characterization and transport properties.

## 2. Materials and Methods

### 2.1. Materials

A commercial, 50% biosourced (reference *PBE003 BB*) polybutylene succinate (PBS) was supplied by Natureplast^©^ (Ifs, France). Graphene nanoplatelets (GnP) with an average of 15 µm in length and a 11–15 nm thickness was provided by SkySpring Nanomaterials^©^ (Houston, TX, USA) under the reference 0544DX Graphene nanopowder.

### 2.2. Film Processing

A detailed procedure was reported in a previous paper by Cosquer et al. [7]. A masterbatch with a theoretical GnP amount of 5 wt.% was first realized. Prior to the melt process, PBS pellets were dried overnight at 80 °C in a vacuum oven and added to an internal mixer, an HAAKE Minilab micro-compounder from Thermo Fisher Scientific^©^ (Waltham, MA, USA) for 2 min at 170 °C. The rotor speed was adjusted at 50 rpm. Then, GnP powder was added and blended for another 3 min in the same conditions. The obtained masterbatch was diluted with neat PBS pellets to obtain the targeted weight percentage. The pellets were extruded using a double screw Micro compounder Xplore^©^ MC 15 HT (Sittard, The Netherlands) at 170 °C for 5 min and then injected into 2 mm thick disks using the injection molder Xplore IM 12 at 150 °C and two steps of pressure: 8 bar for 3 s and 5 bar for 10 s. The obtained disks were hot pressed to form films of about 100 µm thick with a Polystat 200 T of Servitec^©^ (Wustermark, Germany) at 150 °C and 150 bar. PBS/GnP*x* nanocomposites, with “*x*” the theoretical GnP weight percentage ranging from 0.1 to 2, and neat PBS films with a regular thickness of 100 ± 5 µm were obtained.

Theoretical and real compositions (determined by thermogravimetric analysis under air condition) and the sample code of the prepared films are compiled in Table 1. The difference between the theoretical and real compositions are explained by the experimental difficulties encountered during the masterbatch achievement due to the powdering of GnP.

### 2.3. Attenuated Total Reflectance ATR-FTIR

The chemical structure was investigated by IR spectroscopy. IR spectra were recorded with a Nicolet™ iS™5 spectrometer from Thermo Fisher Scientific^©^ (Waltham, MA, USA), in ATR (attenuated total reflection) mode. Each spectrum was recorded at 25 °C with a nominal resolution of 4 cm^−1^ and 32 scans in summation between 4000 and 600 cm^−1^.

### 2.4. Raman Spectroscopy

The Raman spectroscopy measurements were conducted between 10 and 3563 cm^−1^ on the PBS and PBS/GnP nanocomposites using a DXR™ from Thermo Fischer Scientific^©^ (Waltham, MA, USA) and a laser wavelength of 532 nm with a power of 10 mW. The estimated spot size of the measurement was 2.1 µm.

### 2.5. Scattering Electron Microscopy (SEM)

Dispersion of GnP was analyzed by scattering electron microscopy (SEM) with a FEI^©^ QUANTA FEG 250 (Hillsboro, OR, USA) operated at an accelerating voltage of 5 kV and were performed at the ‘‘Centre Technologique des Microstructures’’ of the University Claude Bernard Lyon 1. All samples were prepared cryo-fractured and then covered by a thin layer of carbon.

### 2.6. Differential Scanning Calorimetry

Differential scanning calorimetry (DSC) experiments were carried out with a Q200 apparatus from TA Instrument^©^ (New Castle, DE, USA) under a nitrogen atmosphere following a three step process. Samples were heated from −70 °C to 150 °C with a heating rate of 10 °C.min^−1^. The crystallinity index ($X_{C}$) was calculated using the following equation:

(1) $$X_{C}=\frac{\Delta H_{m}}{{\Delta H}_{m}^{0}\cdot \left(1-\varphi \right)}$$

where *φ* is the mass fraction of GnP added in the nanocomposite; $∆H_{m}$ is the melting enthalpy of the sample$;\;∆H_{m}^{0}$ is the extrapolated value of the enthalpy corresponding to the melting of theoretical 100% crystalline pure PBS, which was taken at 200 J g^−1^ [11,12,13,14]. Each value measured was displayed as the average value on at least three samples.

### 2.7. Dynamic Vapor Sorption (DVS)

Water sorption isotherms of the different films were determined at 10 °C, 25 °C, and 40 °C by using the dynamic vapor sorption analyzer, DVS Advantage from Surface Measurement Systems^©^ (London, UK). Each sample was pre-dried in the DVS Advantage by exposure to dry nitrogen until the equilibrated dry mass was obtained (*m*_0_). A partial pressure of vapor (*p*) was then established within the apparatus by mixing controlled amounts of dry and saturated nitrogen and the mass of the sample (*m_t_*) was followed as a function of time. The mass of the sample at equilibrium (*m_eq_*) was considered to be reached when changes in mass with time (*dm/dt*) were lower than 2.10^−4^ %min^−1^ for at least 5 min. Next, vapor pressure was increased in suitable activity up to 0.9 in steps of 0.1. The value of the mass gain at equilibrium (*M*), defined as $\left(m_{eq}-m_{0}\right)/m_{0}$ for each water activity (*a_w_*), allowed us to plot the water sorption isotherm for each sample.

The solubility coefficient, *S*, was calculated at each water activity according to the following equation:

(2) $$S=\frac{M\times \bar{V_{m}}\times \rho_{PBS}}{M_{w}}\times \frac{1}{p_{vap}\times a_{w}}$$

where *M* is the mass gain at equilibrium for a given water activity; $\bar{V_{m}}$ is the molar volume of water; *ρ_PBS_* is the density of PBS; *M_W_* is the molar weight of water; and *p_vap_* is the vapor pressure of water at the considered temperature. *p_vap_* was calculated with the Antoine equation using the three parameters taken from Yaws et al.,’s Handbook [15].

The sorption rate was also estimated at each water activity by applying Fick’s diffusion law. Considering the film thickness (*L*), the water diffusion coefficient (*D*) was calculated for the short time (*t*) using the following equation:

(3) $$\frac{m_{water\;t}\;}{m_{water\;eq}}=\frac{4}{L}{\left(\frac{D\cdot t}{\pi }\right)}^{0.5}$$

where $m_{water\;t}$ is the mass of water sorbed as a function of the time and $m_{water\;eq}$ is the mass of water sorbed at equilibrium for a given water activity. The accuracy on the values of the water mass gain at equilibrium and the values of the diffusion coefficient were estimated to be better than 5%.

### 2.8. Water Permeation

Water permeability measurements were performed on a Mocon^©^ Permatran W3/33 (Minneapolis, MI, USA) equipped with an infrared sensor at 10 °C, 25 °C, and 40 °C. The detector was calibrated by using polyethene terephthalate (PET) films. The test cell was composed of two chambers separated by a film. Prior to testing, films were conditioned in a nitrogen atmosphere in the unit for at least 12 h to remove traces of atmospheric water vapor. Water molecules in vapor or liquid state were introduced in the upstream compartment of the test cell. Water transferred through the film was conducted by the carrier N_2_ gas to the infrared sensor.

The water permeability coefficient ($P_{H_{2}O}$) was calculated by considering the following equation:

(4) $$P_{H_{2}O}=\frac{J_{st_{H_{2}O}}\cdot L}{\Delta p}$$

where *L* is the thickness of the film; $J_{st_{H_{2}O}}\;$is the water stationary flux; and ∆p is the difference of pressure between the upstream and the downstream compartments of the permeation cell. $P_{H_{2}O}$ values were expressed in Barrer (1 Barrer = 10^10^ ${\mathrm{cm}}_{\mathrm{STP}}^{3}$ cm cm^2^ s^−1^ cm_Hg_^−1^ = 3.36 × 10^−16^ mol m^−1^ m^−2^ s ^−1^ Pa^−1^) and the precision of the obtained values was estimated to be better than 5%. $P_{H_{2}O}$ was determined at different water activity: *a_w_* = 0.5, 0.7, 0.8, and 1.

### 2.9. Dioxygen Permeation

Dioxygen (O_2_) permeability measurements were performed on a Mocon^©^ Oxtran 2/21 (Minneapolis, MI, USA) equipped with a colorimetric sensor at 10 °C, 25 °C, and 40 °C. The test cell was composed of two chambers separated by the film. Nitrogen containing 2% of hydrogen (N_2_/H_2_) was used as the carrier gas and pure dioxygen was used as the test gas. The water activity of the two gases was controlled by a humidifier. Prior to testing, films were conditioned in a N_2_/H_2_ atmosphere in the unit for at least 12 h on the one hand to remove traces of atmospheric dioxygen, and on the other hand, to be at the water uptake equilibrium condition of the film. Next, dioxygen was introduced in the upstream compartment of the test cell. O_2_ molecules transferred through the film were conducted by the carrier N_2_/H_2_ gas to the colorimetric sensor. A steady-state line was obtained after a transitory state.

The dioxygen permeability coefficient ($P_{O_{2}}$) was calculated considering the following equation:

(5) $$P_{O_{2}}=\frac{J_{st_{O_{2}}}\cdot L}{\Delta p}$$

where *L* is the thickness of the film; $J_{st_{O_{2}}}\;$is the dioxygen stationary flux; and ∆p is the difference of pressure between the upstream and the downstream compartments of the permeation cell. $P_{O_{2}}$ values were expressed in Barrer (1 Barrer = 10^10^ ${\mathrm{cm}}_{\mathrm{STP}}^{3}$ cm cm^2^ s^−1^ cm_Hg_^−1^ = 3.36 × 10^−16^ mol m^−1^ m^−2^ s^−1^ Pa^−1^) and the precision of the obtained values was estimated to be better than 5%. $P_{O_{2}}$ was determined at different water activity: *a_w_* = 0.5, 0.7, 0.8, and 1.

## 3. Analysis

### 3.1. Thermodynamic Analysis: Isotherm Modeling

The sorption isotherms can be mathematically described in order to obtain useful information regarding the sorption mode and the type of interactions involved throughout the sorption process. Numerous models are available in the literature for the description of the water vapor sorption in polymers. The Guggenheim, Anderson, and de Boer (GAB) equation is one of the more widely used equations for the fitting of BET type II and BET type III [16,17,18]. The GAB equation describing the isotherms assumes localized physical adsorption in multilayers with no lateral interactions. According to this theory, the first molecules are sorbed very strongly in the monolayer. Once the monolayer is reached, the molecules will subsequently be sorbed with weaker interaction, with the sorbent surface. Moreover, the range in energy levels is between those of the monolayer molecules and the bulk liquid. The equation of the GAB model introduces three physical parameters (*M_m_*, *C_G_*, and *K*) and defines the mass gain as:

(6) $$M=M_{m}\frac{C_{G}\cdot K\cdot a_{w}}{\left(1-K\cdot a_{w}\right)\left(1+\left(c_{G}-1\right)\cdot K\cdot a_{w}\right)}$$

where *M_m_* is the monolayer value and characterizes the availability of active sites for permeant molecules by the polymer; *C_G_* is the Guggenheim constant depicting the strength of the binding of water to the primary binding sites; and *K* is a correction factor, since it corrects the properties of the multilayer molecules relative to the bulk liquid (when *K*~1, the properties of the multilayer molecules are similar to bulk liquid water) [19]. To evaluate the accuracy of the GAB model to describe the water sorption isotherms of the studied films, the mean relative percentage of the deviation modulus (*MRD*) is commonly used and defined as:

(7) $$MRD\left(\%\right)=\frac{100}{N}\overset{N}{\underset{i=1}{\sum }}\frac{\left|m_{i}-m_{pi}\right|}{m_{i}}$$

where *m_i_* is the experimental value; *m_pi_* is the predicted value; and *N* is the number of experimental data.

The mean relative percentage deviation modulus (*MRD*) is a criterion widely used in the literature and a modulus value below 10% indicates a good fit for practical purposes [20]. The GAB parameters were determined by fitting each isotherm with OriginLab software.

The high increase of uptake observed for BET II and BET III isotherms at high activities is generally explained by a clustering phenomenon. Zimm and Lundberg developed a method, centered on the basis of statistical mechanisms that analyze this phenomenon from the shape of the experimental isotherm [20]. Neglecting the isothermal compressibility of the polymer–permeant solution makes the free energy function of the system essentially dependent upon the first derivative of the activity with respect to the permeant volume fraction. The elaborated relation appears as follow:

(8) $$\frac{G_{S}}{V_{w}}=-\left(1-\phi_{w}\right){\left[\left(\frac{\delta \left(\frac{a_{w}}{\phi_{w}}\right)}{\delta a_{w}}\right)\right]}_{P,T}-1$$

where *G_s_* is the cluster integral; *V_w_*. and *φ_w_* are the partial molecular volume of the penetrant and the volume fraction of the penetrant, respectively. A *G_S_/V_w_* value equal to −1 indicates that water dissolves into the polymer matrix randomly, instead at higher values, *G_S_/V_w_* > 1 means that the concentration of water in the neighborhood of a given water molecule is greater than the average concentration of the water molecules in the polymer. The quantity *G_S_/V_w_* is the mean number of molecules in excess of the mean concentration of water in the neighborhood of a given molecule [21]. Thus, the mean cluster size (*MCS*) can be evaluated by the following equation:

(9) $$MCS=1+\left(\frac{\phi_{w}\cdot G_{S}}{V_{w}}\right)$$

*MCS* values can be calculated from the GAB parameters considering the following equation:

(10) $$MCS=\frac{{\left(\rho_{w}/\rho_{p}\right)}^{2}}{M^{2}{\left(1+\frac{\rho_{w}/\rho_{p}}{M}\right)}^{2}}\times \left[1-\frac{M}{M_{m}\times C_{G}}\left(-2\cdot K\cdot a_{w}(C_{G}-1\right)-2+C_{G})\right]$$

where *ρ_w_* is the water density and *ρ_p_* is the density of the polymer (PBS = 1.18 g.cm^−3^ [11]). *Mm*, *C_G_*, and *K* are the three GAB parameters as explained above and *M* is the mass gain at equilibrium.

### 3.2. Temperature Dependency on Transport Properties

Permeation is a solution-diffusion process that can be described in terms of the transport and sorption coefficient for every polymer–gas system. The permeability coefficient can thus be expressed as the product of the diffusion coefficient and solubility coefficient values [22]:

(11) P=D×S

with *P* is the permeability coefficient; *D* is the diffusion coefficient; and *S* is the solubility coefficient. *D*, the diffusion coefficient, is a kinetic term governed by the amount of energy necessary for a penetrant to execute a diffusive jump through the polymer and the intrinsic degree of segmental packing in the matrix [23]. *S*, the solubility coefficient, is a thermodynamic term that depends on various factors such as interactions between the permeant and the polymer, the condensability of the penetrant, and the amount of penetrant-scale non-equilibrium voids present in the material [24].

For the temperature ranges selected in a region where there is no transition in the polymer (e.g., glass transition) and penetrating gas (e.g., boiling point), the permeation of gas through the polymer against temperature follows an Arrhenius equation, as for the diffusion and solubility [22,25]:

(12) $$P=P_{0}\times \exp\left(\frac{-E_{p}}{RT}\right)$$

(13) $$D=D_{0}\times \exp\left(\frac{-E_{d}}{RT}\right)$$

(14) $$S=S_{0}\times \exp\left(\frac{-\Delta H_{s}}{RT}\right)$$

where *P*_0_, *D*_0_, and *S*_0_ are pre-exponential factors; *E_p_* is the apparent activation energy for the permeation process; *E_d_* is the activation energy for the diffusion process; and Δ*H_s_* is the heat of the sorption of water into the polymer. By combining Equations (12)–(14), it appears that the apparent activation energy for permeation is the sum of the activation energy for diffusion and the heat sorption for a particular penetrant:

(15) $$E_{p}=E_{d}+\Delta H_{s}$$

This relation can thus be used to calculate the theoretical value of *E_p_* from the experimentally obtained values of *E_d_* and Δ*H_s_*. The heat of sorption, Δ*H_s_*, can be calculated by the following equation:

(16) $$\Delta H_{s}=\Delta H_{cond}+\Delta H_{l}$$

where Δ*H_cond_* is the molar heat of condensation and is always negative [26]. Δ*H_l_* is the partial heat of mixing.

For gases where the studied temperature (*T_study_*) is well above their critical temperature (*T_crit_*), the temperature after which the gas cannot be liquified by compression such as N_2_, H_2_, O_2_, etc., the Δ*H_cond_* term is very weak and, thus Δ*H_s_* is governed by Δ*H_l_*. Since the Δ*H_l_* values are small and positive for permanent gas, Δ*H_s_* will be positive, and the solubility coefficient will increase slightly by increasing the temperature. In the case of more condensable gases (in which *T_crit_* > *T_amb_*) such as CO_2_, SO_2_, NH_3_, H_2_O, etc., the Δ*H_cond_* term has a strong contribution compared with the Δ*H_l_* and will lead to a negative value of Δ*H_s_*. Hence, the solubility coefficient will decrease when the temperature increases [21,27].

## 4. Results and Discussion

### 4.1. Morphology

In this study, the morphology was further investigated. The chemical structure of the PBS, PBS/GnP, and GnP structures were studied using FTIR-ATR and Raman spectroscopy analyses.

**Figure 1:**
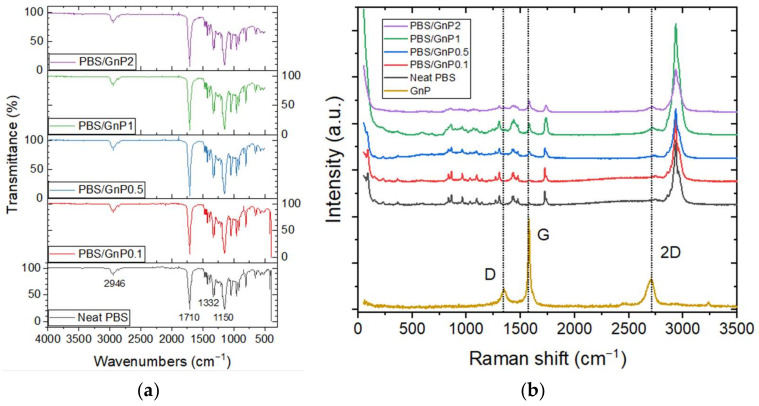
(**a**) FTIR-ATR spectra of the neat PBS and PBS/GnP nanocomposites and (**b**) the Raman spectra of the neat PBS and PBS/GnP nanocomposites.

The FTIR-ATR spectra of neat PBS and PBS/GnP films are presented in Figure 1a. The chemical structure of neat PBS was confirmed by the presence of the typical main absorption peaks such as C–H bond stretching at 2946 cm^−1^, carbonyl C=O stretching vibrations at 1710 cm^−1^, -COO-bond stretching at 1332 cm^−1^, C–O–C stretching at 1150 cm^−1^, with respect to the literature [13,28]. No modification was observed in the chemical structure of PBS after the addition of GnP into the matrix. Raman spectroscopy measurements were also performed complementary to FTIR-ATR and the spectra are displayed in Figure 1b. The GnP powder displayed the typical spectrum of a graphite-like material defined by the 2D band at 2710 cm^−1^, the G band at 1580 cm^−1^, and the D band at 1350 cm^−1^ [29]. The number of graphene layers can be monitored using the position of the 2D band [30]. A shift to a lower Raman shift is a characteristic of an exfoliation of graphene layers. The position of the 2D band remained unchanged, regardless of the weight percentage of GnP into the nanocomposite. The intensity of all three peaks of GnP seemed to increase when the GnP amount increased in the nanocomposites.

**Figure 2:**
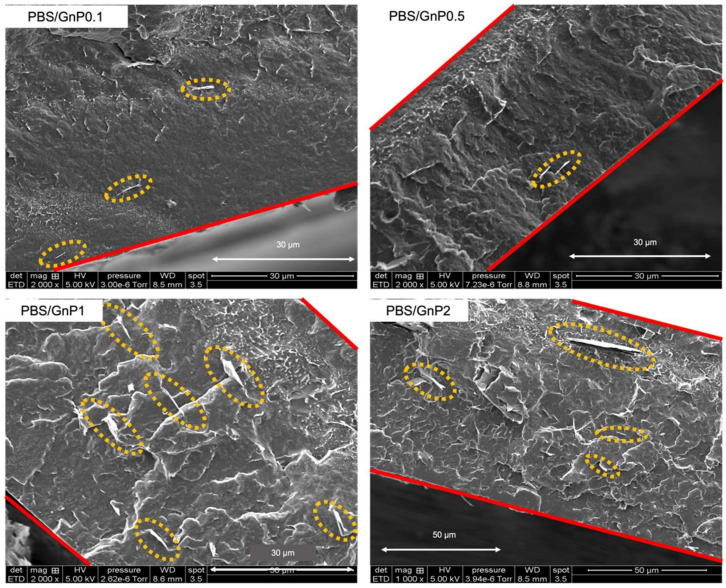
The scattering electron microscopy images of the nanocomposite films.

SEM was used to investigate the state of the GnP nanoplatelet dispersion in the PBS matrix. The images are shown in Figure 2.

A similar dispersion level was found, regardless of the amount of GnP (GnP can be seen in the yellow circles). Considering the initial particle/aggregate size (average of 25 µm, measured by scanning electronic microscopy) (Supplementary Materials Figure S1), the dispersion of GnP into the PBS matrix can be considered as good and it revealed good interactions of these GnPs with the polymer. Some aggregates were observed for the highest GnP content. For the purpose of clarity, the film edge is marked with red lines. From the SEM micrographs analysis, it appeared that the large majority of GnPs were parallelly oriented to the membrane surface, and thus will theoretically improve the barrier properties, because the barrier properties are known to be dependent of the filler orientation [31].

To obtain information relating to the chain mobility of the amorphous phase and the crystalline phase of the films, the films were also analyzed by differential scanning calorimetry (DSC). The values of the glass transition temperature of the neat matrix and different nanocomposites are listed in Table 2. The obtained values for PBS were found to be equal to −35 °C. Bhatia et al., found a similar value of *T_g_*, around −34 °C [32]. By considering uncertainty on each value, no significant change in the *T_g_* of the PBS matrix was observed after the incorporation of GnP.

The estimated crystallinity index (*X_c_*) of the neat PBS was 38%. No modification of *X_c_* was observed when the GnPs were added. A similar phenomenon was found by Goncalves et al., in polylactic acid (PLA)/GnP nanocomposites showing a non-significant decrease of *X_c_* in the nanocomposites [33]. Accordingly, the GnP fillers had no effect on the crystallization of the PBS matrix.

### 4.2. Morphology

**Figure 3:**
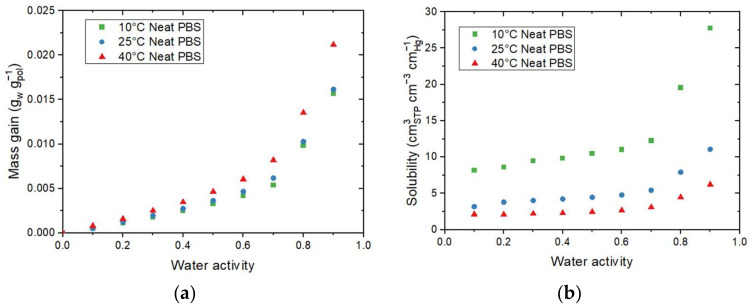
Evolution as a function of the water activity for the neat PBS of (**a**) the mass gain (M) and the average number of water molecules sorbed in a single amorphous unit of polymer (N_i_) and (**b**) the solubility coefficient at 10 °C, 25 °C, and 40 °C.

The sorption isotherms at 10 °C, 25 °C, and 40 °C for neat PBS are displayed in Figure 3a. All isotherm curves displayed a BET III shape according to the classification of Brunauer–Emmett–and Teller (BET), regardless of the temperature of the isotherm [34]. It consists of a linear evolution of water uptake at low water activity (*a_w_* ≤  0.4), followed by a convex part at higher activity (*a_w_* > 0.6). The increase in water uptake at high activity is usually explained by the formation of water clusters [35]. It can be found in Figure 3a that the mass gain slightly increased when the temperature increased. Values at 10 °C and 25 °C were close for the same water activity while values at 40 °C were always slightly higher across the whole range of the tested water activity.

Since the crystalline part of the PBS matrix is considered as impermeable to the water molecules, the mass gain at equilibrium (*M_a_*) was calculated as a function of the amorphous phase of the polymer matrix using the crystalline index determined from the DSC analysis. From the mass gain data, the average number of water molecules sorbed in a single amorphous unit of polymer (*N_i_*) was calculated as follows:

(17) $$N_{i}=M_{a}\cdot \frac{M_{p}}{M_{w}}$$

where *M_a_* is the mass gain at equilibrium of PBS amorphous part; *M_p_* and *M_w_* are the molar mass of the studied polymer unit (*M_p_* = 172 g mol^−1^) and the molar mass of water (*M_w_* = 18 g mol^−1^), respectively.

The evolution of *Ni* as a function of the water activity is presented on the right Y-axis in Figure 3a. The obtain *Ni* curves logically showed the same shape as the isotherm curves. From this representation, it can be seen than for *a_w_* = 0.9, there was one water molecule on average sorbed every 4 units of PBS in the amorphous phase for 10 °C and 25 °C, while there was one water molecule on average sorbed every 3 units of PBS in the amorphous phase at 40 °C.

The evolution of the solubility for 10 °C, 25 °C, and 40 °C as a function of the water activity are shown in Figure 3b. The solubility coefficient values slightly increased until *a_w_* = 0.6 and drastically increased for the high water activity regardless of the temperature. The increase in the solubility at high water activity can be explained by the clustering effect described previously. The water–water interactions are greater than the water–polymer interactions, resulting in an increase in the free volume, hence an increase in the solubility coefficient. This evolution agreed with the BET III isotherm shape of the curve. Contrary to the tendency observed for mass gain at equilibrium, the solubility coefficient values decreased as the temperature increased, in accordance with the study of Siparsky et al., on PLA [9]. This result shows the typical behavior of condensable gases such as water permeating through the polymer [9] and can be explained by the fact that both concentration (*C*) and partial pressure (*p_vap_*) used for the determination of *S* increased as the temperature increased. However, the evolution of *p_vap_* was more important than the evolution of *C*, resulting in a decrease in *S* as the temperature increased.

The solubility coefficients of water vapor in PBS at 25 °C, between 2.5 and 10$\;{\mathrm{cm}}_{\mathrm{STP}}^{3}\cdot {\mathrm{cm}}^{-3}{\;\mathrm{cm}}_{\mathrm{Hg}}^{-1}$ dependening on the water activity, were compared with those of other polymers. The values of *S* were comparable to hydrophobic polymers such as polyethylmethacrylate (PEMA), polycaprolactone (PCL), and polylactic acid (PLA), which presented a value of *S* equal to 2.7, 2.1 and 5.3 ${\mathrm{cm}}_{\mathrm{STP}}^{3}{\;\mathrm{cm}}^{-3}{\;\mathrm{cm}}_{\mathrm{Hg}}^{-1}$, respectively at 25 °C [9]. The obtained values of *S* highlight the hydrophobic character of PBS regarding other hydrophilic polymers such as cellulose nitrate, polyethyl cellulose, and polymide (Kapton), which presented values of 23.4, 31.2, and 28.5 ${\mathrm{cm}}_{\mathrm{STP}}^{3}{\;\mathrm{cm}}^{-3}{\;\mathrm{cm}}_{\mathrm{Hg}}^{-1}$, respectively, at 25 °C [9].

**Figure 4:**
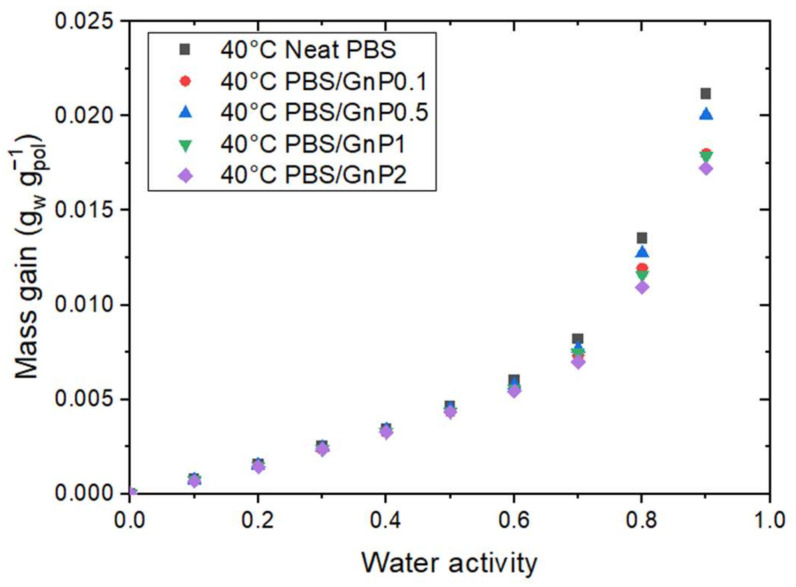
The evolution of the mass gain as a function of the water activity at 40 °C for neat PBS and the corresponding nanocomposites.

In a previous study, Cosquer et al., discussed the effect of GnP addition on the water sorption of PBS at 25 °C [7]. The author concluded on the insignificant effect of the presence of GnP on the water sorption capacity (mass gain) of the nanocomposites. In this study, the effect of temperature on the PBS/GnP nanocomposites were investigated at two other temperatures of 10 °C and 40 °C. Considering that the crystalline part of the PBS matrix and GnP are both impermeable to water molecules, the mass gain at equilibrium was calculated as a function of the amorphous phase of the polymer matrix using the crystalline index determined from the DSC analysis. The evolution of the mass gain in the PBS amorphous phase as a function of the water activity at 40 °C for the neat PBS and the PBS/GnP nanocomposites are presented in Figure 4. The mass gain data for 10 °C and 25 °C can be seen in Supplementary Materials Figure S2 and solubility data at all three temperatures in Supplementary Materials Figure S3. From Figure 4, it should be noted that regardless of the temperature and GnP content, all isotherm curves displayed a BET III shape [34]. The addition of GnP did not lead to a significant change in the water sorption capacity of the films. A single curve was obtained, confirming that the presence of GnP had no impact on the water sorption mechanism since the water sorption phenomenon occurred in the amorphous part of the PBS matrix. Nevertheless, this result confirms that the interfaces between the matrix and the nanofillers were not diffusive, suggesting a good interfacial adhesion.

To go deeper into the understanding of the sorption process as a function of the temperature, sorption isotherms were modeled using the GAB equation (Equation (6)). The three parameters defined in the GAB model are listed in Table 3 for each temperature.

First, the examination of *MRD* indicated that the model was convenient, allowing for an accurate description of the experimental sorption isotherm. For all formulations, the *M_m_* values linearly increased when the temperature increased. The *C_g_* and *K* values did not depend either on the temperature or the filler content. Thus, the analysis of the GAB parameters of the water sorption process led to the conclusion that the main difference observed was the amount of water molecules sorbed in the monolayer, which depend on the studied temperature.

**Figure 5:**
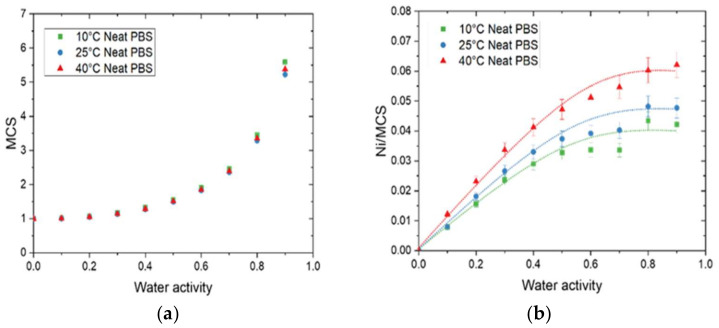
The evolution as a function of water activity for neat PBS of (**a**) the mean cluster size (MCS) and (**b**) the average number of sorption sites per monomer unit in the amorphous phase (*N_i_/MCS*) at 10 °C, 25 °C, and 40 °C.

By using the theory by Zimm and Lundberg, it was possible to determine the *MCS* (Equation (10)) values from the parameters deduced from the GAB equation for the neat matrix and nanocomposites films at 10 °C, 25 °C, and 40 °C (Equation (6)). The plot of *MCS* versus the water activity for the neat matrix at different temperatures is represented in Figure 5a.

As the same curve was obtained in Figure 5a for all temperatures, the number of water molecules per sorption site (*MCS*) remained almost constant. The *MCS* values were close to unity at low water activity (below *a_w_* = 0.4) and then increased at higher water activities. As explained by Cosquer et al., beyond *a_w_* = 0.4, interactions for a water molecule to another sorbed molecule appeared and became preponderant, leading to the progressive formation of water clusters [7].

The *N_i_/MCS* values, corresponding to the average number of sorption sites per monomer unit in the amorphous phase, were calculated and the evolution as a function of water activity is plotted in Figure 5b. The curves exhibited the same shape: a linear increase at low water activity and then a plateau was reached. As described by Sabard et al., the increase in *N_i_/MCS* can be explained by an individual distribution of the water molecules on the different monomer units of PBS in the amorphous phase whereas the plateau is due to clustering phenomenon [36]. The value of *N_i_/MCS* at the plateau increased as the temperature increased and its value was equal to 0.042, 0.047, and 0.062 for 10 °C, 25 °C, and 40 °C, respectively. Hence, the average number of sorption sites was one every 24, 21, and 16 monomer units of PBS in the amorphous phase for 10 °C, 25 °C, and 40 °C, respectively. This result is coherent with the increase in *M_m_* and can be explained by an increase in chain mobility when the temperature increased. In the presence of GnP, regardless of the amount of filler, a similar tendency was obtained in comparison with the neat matrix (Supplementary Materials S5).

The solubility coefficient is a thermodynamic term and follows an Arrhenius law. The molar heat of sorption can thus be calculated from the solubility data.

**Figure 6:**
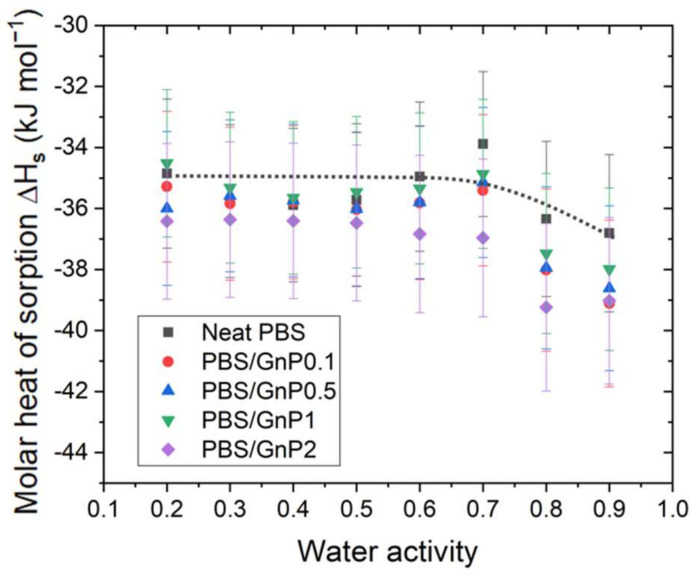
The evolution of the molar heat of sorption as a function of the water activity calculated from the Arrhenius plot with Equation (14) for the neat PBS and PBS/GnP nanocomposites.

The Δ*H_S_* was calculated for each activity for the neat PBS and PBS/GnP nanocomposites from Equation (14) and plotted as a function of the water activity (Figure 6). In each case, the correlation factor (R^2^) for the calculation of Δ*H_S_* was higher than 0.99 (Supplementary Materials Figure S6).

Figure 3 shows the hydroxybutyrate] (PHB), poly(ε-caprolactone) (PCL), in the temperature range from 36 to 60 °C and the obtained values of Δ*H_S_* were −27.4, −48.4, −46.8, and −43.4 kJ mol^−1^, respectively [37]. However, the water activity used to perform the analyses was not reported in the study by Yoon et al. [37]. In our study, the Δ*H_S_* for the neat PBS was constant until *a_w_* >0.7 with a value around −35 kJ mol^−1^ and then decreased for higher activities to reach a value around −37 kJ mol^−1^. This decrease was attributed to the clustering of water molecules and thus the condensation of water in the PBS matrix. Siparsky et al., measured the heat of sorption of water in PLA and found it to be −40 kJ mol^−1^ [9]. They presented this result as evidence of the water-cluster model because this value was close to the heat of the condensation of water.

After the addition of filler, regardless of the amount of GnP, the evolution curves of Δ*H_S_* as a function of the water activity presented the same shape than that obtained for the PBS matrix. Considering the uncertainty, no significant modification in the Δ*H_S_* values were seen, and no clear trend was observed between the GnP amount and Δ*H_S_* values.

**Figure 7:**
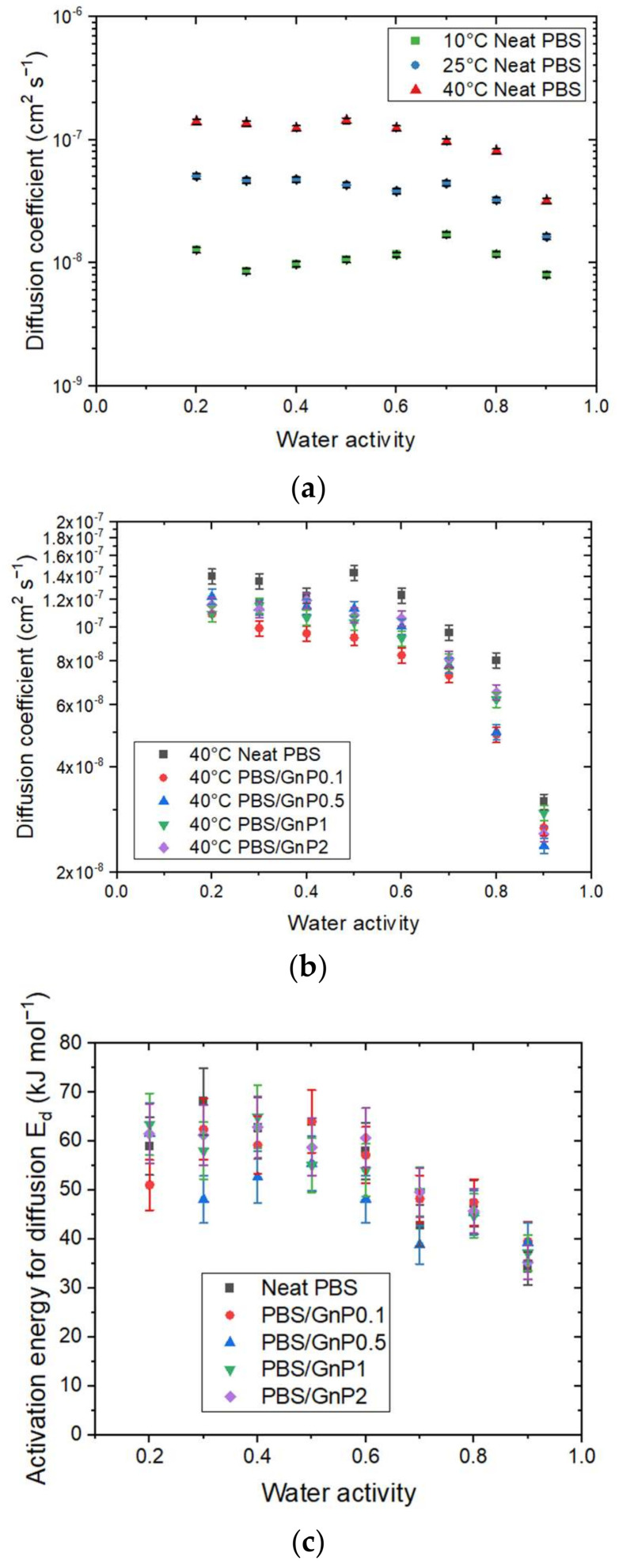
The evolution as a function of the water activity of (**a**) the diffusion coefficient at 10 °C, 25 °C, and 40 °C of the neat PBS, (**b**) diffusion coefficient at 40 °C of the neat PBS and PBS/GnP nanocomposites, and (**c**) the activation energy for diffusion E_d_ for the neat PBS and PBS/GnP nanocomposites.

The kinetics of water sorption in the neat PBS was also investigated and the evolution of the water diffusion coefficients at different temperatures as the function of water activity are plotted in Figure 7a.

The *D* value was constant up to a water activity of 0.6 and decreased for higher water activity. This evolution of *D* was in accordance with the sorption isotherm shape of the sorption isotherm curve. The constant value of *D* was explained by Henry’s sorption mode and the decrease was attributed to the water clustering phenomenon. Moreover, for each water activity, *D* increased as the temperature increased. This effect may be explained in terms of an increase in the free volume directly related to the expansion of the polymer due to the enhancement of the segmental motions. Thus, the diffusion process of molecules is facilitated.

The diffusion coefficient at 40 °C for the neat PBS and PBS/GnP nanocomposites (data for 10 °C and 25 °C are available in Supplementary Materials Figure S7) are presented in Figure 7b. Focusing on the impact of the GnP on the water diffusion properties of PBS, it appears that adding GnP fillers tends to reduce the diffusion coefficient. However, it was difficult to see any trend with the amount of GnP. In a previous study, Cosquer et al., showed that at 25 °C, *D* seemed to decrease slightly as the amount of GnP increased [7]. This could be principally explained by a tortuosity effect in the PBS matrix, which led to an increase in the diffusion path and thus the diffusion rate of water molecules in the neat PBS [8,38].

The activation energy of diffusion (*E_d_*) was calculated from Equation (13) for each water activity. In each case, the correlation factor (R^2^) of the Arrhenius plot was higher than 0.99 (Supplementary Materials Figure S10). Flaconnèche [27] explained that *E_d_* represents the energy level that a molecule should reach to make a jump between one position and another. This value is always positive. Then, the *E_d_* value is as high as the cohesive forces between chains are strong. For neat PBS, *E_d_* was constant until a value of *a_w_* > 0.6 with a value around 60 kJ mol^−1^ and then decreased to reach a value at *a_w_* = 0.9 equal to 34 kJ mol^−1^. The decrease in *E_d_* for high water activity can be explained by a decrease in the cohesive energy density of the polymer due to the formation of water clusters, as seen previously. In other terms, a decrease in *E_d_* indicates that water molecules must overcome lower intermolecular forces within the polymer to diffuse.

The *E_d_* values of PBS obtained in our study was in the same range order compared to different polyester polymers such as polylactic acid (PLA) [9], polycaprolactone (PCL) [9], and polyethylene terephthalate (PET) [39], which presented for *a_w_* = 0.9 a value of *E_d_* of 37, 31, 44 kJ mol^−1^, respectively. A value of *E_d_* equal to 20 kJ mol^−1^ was reported by Siparsky et al., for a polymer blend PLA/PEG (80/20 wt.%) [9]. This lower value can be explained by the presence of PEG, which led to an increase in the polymer chain mobility associated with an increase in the free volume and a decrease in the cohesive energy density.

After the addition of GnP, the evolution of *E_d_* as a function of the water activity presented the same trend as that for the neat matrix. Similarly, to Δ*H_S_*, considering the uncertainty, there was no significant modification in the *E_d_* values of the neat PBS through the addition of GnP in that range of GnP concentration across the whole range of activity. Additionally, there was no clear trend between the GnP amount and the reduction in the *E_d_* values.

### 4.3. Water Permeability

**Figure 8:**
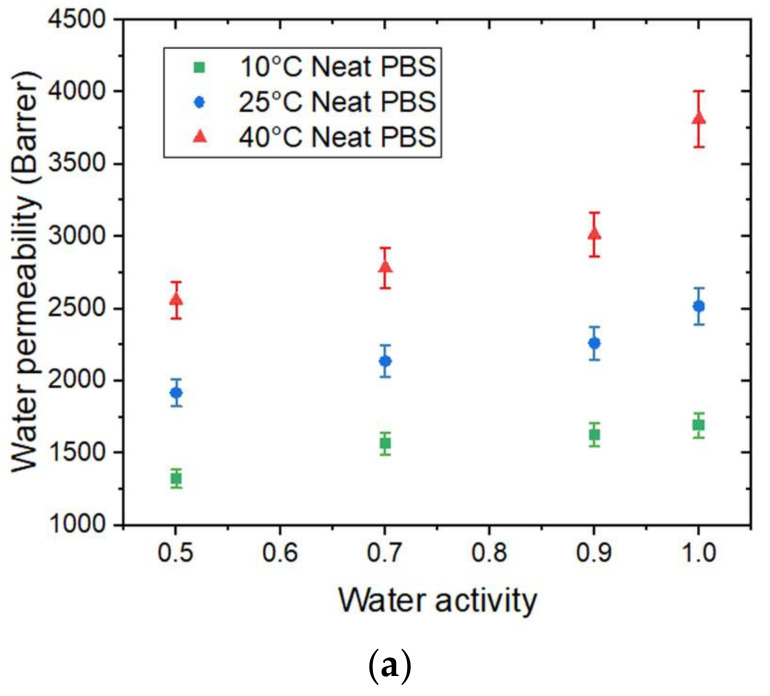

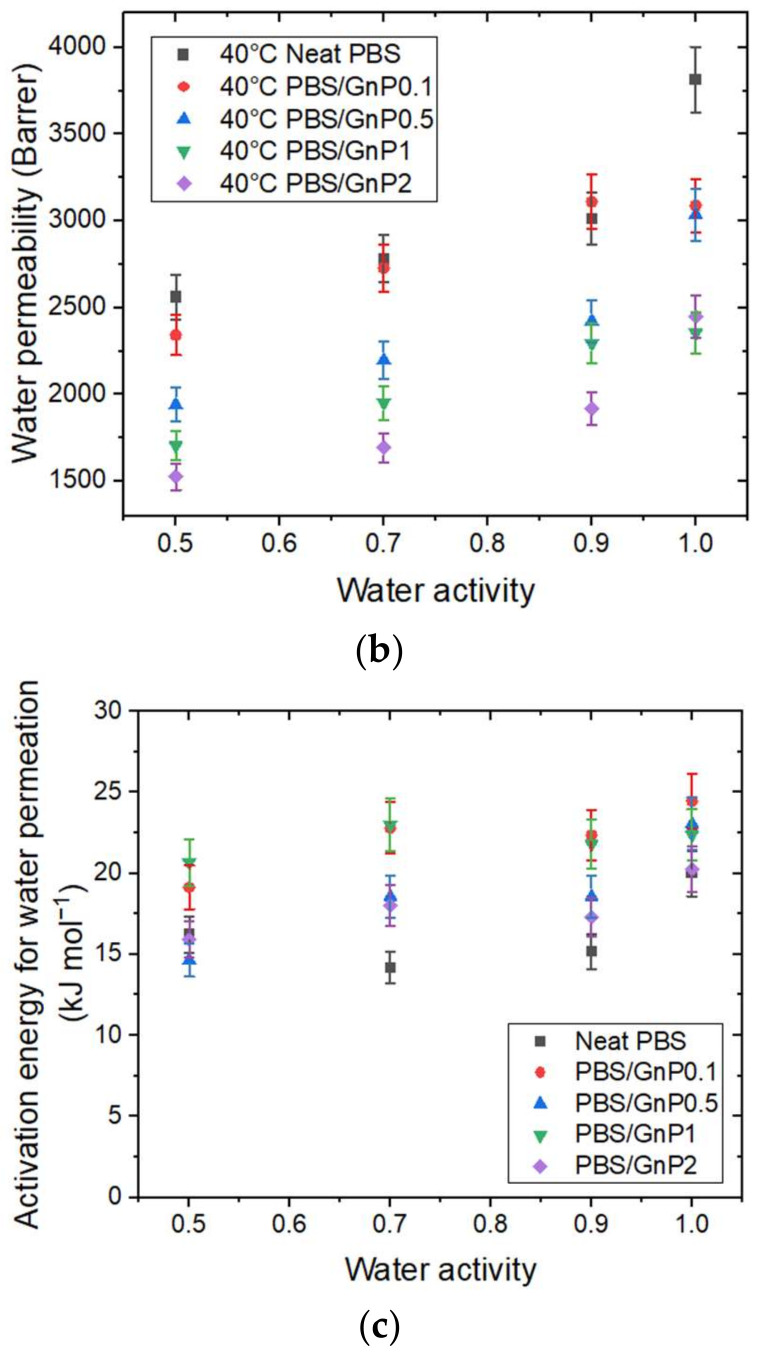
The evolution as a function of the water activity of (**a**) the water permeability coefficient at 10 °C, 25 °C, and 40 °C of the neat PBS. (**b**) The water permeability coefficient at 40 °C for the neat PBS and PBS/GnP nanocomposites and (**c**) the apparent activation energy for water permeation for the neat PBS and PBS/GnP nanocomposites.

The evolution of water permeability ($P_{H_{2}O})\;$of the neat PBS as a function of water activity is presented in Figure 8a.

$P_{H_{2}O}$ increased as the temperature increased. This can be explained by the enhancement of segmental movements of the polymer. The permeability coefficient is given as the product of the solubility coefficient and diffusion coefficient. In our case, the solubility decreased while the diffusivity increased as the temperature increased. The change in water permeability thus depend on the relative changes in *D* and *S*. Here, the permeability coefficient increased as the temperature increased. Thus, the diffusivity was the dominant phenomenon in the permeation process in that case.

In the tested range of water activity, $P_{H_{2}O}$ increased as the water activity increased regardless of the temperature. This result can be explained by the presence of water molecules sorbed by the polymer, which favor the water permeation process.

The evolution of water permeability of the neat PBS and corresponding nanocomposites at 40 °C as a function of the water activity are presented in Figure 8b and the evolutions for 10 °C and 25 °C can be seen in Supplementary Materials Figure S8. Regardless of the temperature, the introduction in GnP led to a decrease in the permeability compared to the PBS matrix across the whole range of water activity. The improvement in the barrier properties increased as the GnP amount increased within the PBS matrix. An improvement of 40% was obtained for the higher GnP content. As reported in the literature, GnP is considered as an impermeable filler for small molecules [40,41]. Furthermore, as shown by the SEM micrographs (Figure 2), a good dispersion as well as a preferential orientation of GnP within the matrix were observed, leading to a significant increase in the gas pathway by a tortuosity effect.

The activation energies for permeation ($E_{p\left(H_{2}O\right)}$) were calculated from the Arrhenius equations. All of the obtained correlation factor values (*R^2^*) of the Arrhenius plot were higher than 0.99 (Supplementary Materials Figure S10). The evolutions of $E_{p\left(H_{2}O\right)}\;$as a function of water activity for the neat PBS and PBS/GnP nanocomposites are presented in Figure 8c. For the neat PBS, considering the uncertainty, the $E_{p\left(H_{2}O\right)}$ values remained almost constant (neat PBS: $\;E_{p\left(H_{2}O\right)}=$15 ± 3 kJ mol^−1^) as the water activity increased. Samaniego-Esguerra et al., obtained $E_{p\left(H_{2}O\right)}\;$values on low density polyethylene (LDPE) that varied from 8 to 15 kJ mol^−1^ over a water activity range from 0.55 to 0.9 for a temperature range between 20 °C and 40 °C [42]. The same authors observed the opposite tendency for $E_{p\left(H_{2}O\right)}$ values of PET with a decrease from 5.6 to 3.4 kJ mol^−1^ for the same range of water activity and temperature. Therefore, it is difficult to conclude on the evolution of $E_{p\left(H_{2}O\right)}$ as a function of the water activity. Once again, by considering the uncertainty, after the addition of GnP, there was no significant modification of $E_{p\left(H_{2}O\right)}$ in the tested range of water activity compared to those obtained for the neat matrix. Additionally, there was no clear trend between the GnP amount and the obtained *E_p_* values.

In this study, the tortuosity effect induced by the GnP was the main reason to explain the improvement in the barrier properties since there was no significant impact of the GnP on the *E_D_*, Δ*H_S_*, and $E_{p\left(H_{2}O\right)}$ for the nanocomposites compared to the neat PBS.

The $E_{p\left(H_{2}O\right)}$ values of the PBS/GnP nanocomposites determined from the water permeation analyses were compared to the *E_p_ H_2_O* values calculated from the *E_D_* and Δ*H_S_* obtained from the water vapor sorption analyses (Equation (15)) and are displayed in Table 4 as an example for *a_w_* = 0.5. Considering the uncertainty, close values were obtained, allowing us to validate this equation.

### 4.4. Dioxygen Permeability

**Figure 9:**
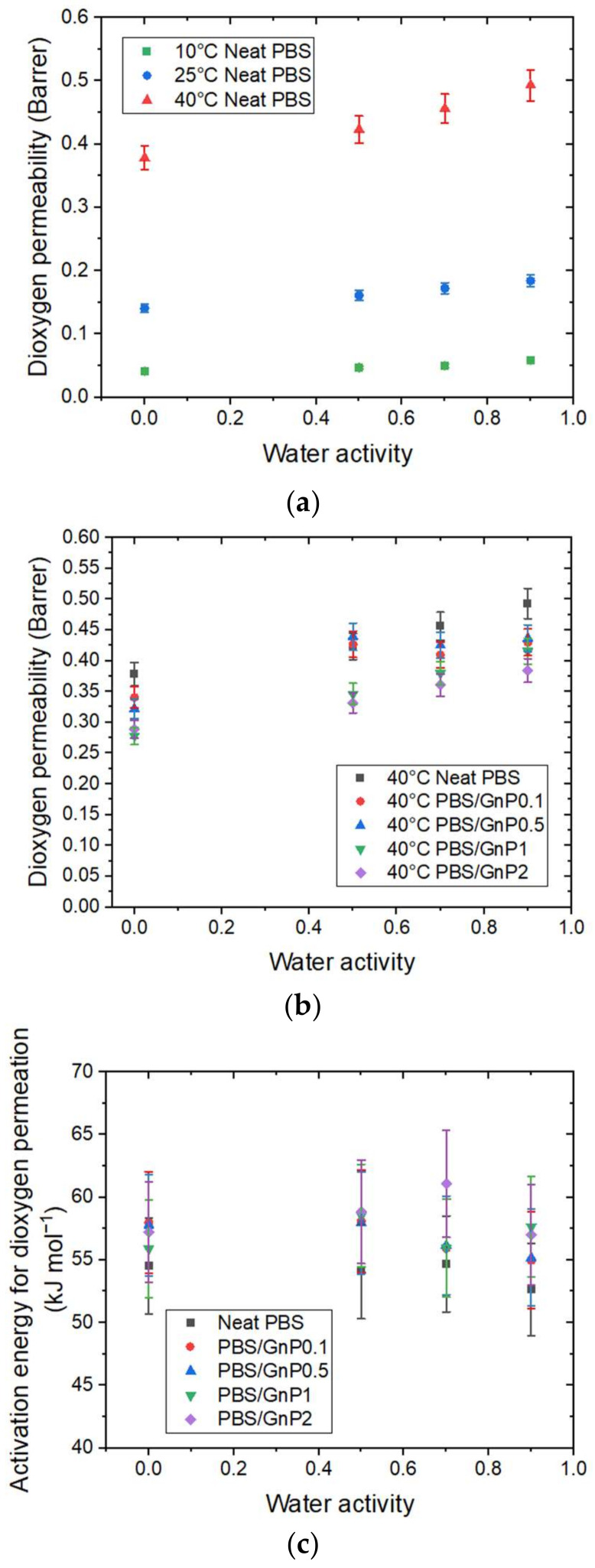
The evolution as a function of the water activity of (**a**) the dioxygen permeability coefficient at 10 °C, 25 °C, and 40 °C for neat matrix. (**b**) The relative permeability coefficient at 40 °C for the neat PBS and PBS/GnP nanocomposites and (**c**) the activation energy for dioxygen permeation $E_{p\left(O_{2}\right)}$ for the PBS and PBS/GnP nanocomposites.

Dioxygen permeability measurements were performed on the neat matrix and different nanocomposites for a water activity range from 0 to 0.9 at 10 °C, 25 °C, and 40 °C. The evolution of the dioxygen permeability coefficient ($P_{O_{2}}$) as a function of the water activity for the different temperatures is shown in Figure 9a.

A significant increase in $P_{O_{2}}$ as the temperature increased can be observed for the neat matrix with a $P_{O_{2}}$ value from 0.041 Barrer at 10 °C to 0.378 Barrer at 40 °C, at an anhydrous state. As seen previously, this increase in permeability as a function of the temperature can be explained by an enhancement of the polymer chain mobility associated with an increase in the free volume. As in the case of the water permeability analysis, in the tested range of water activity, at a given temperature, $P_{O_{2}}\;$increased linearly as the water activity increased and can be explained by the presence of water molecules sorbed, which favors the dioxygen permeation process.

As observed for water permeation, the introduction of GnP led to an improvement in the dioxygen barrier properties. The evolution of $P_{O_{2}}$ for the neat PBS and corresponding nanocomposites at 40 °C as a function of water activity are presented in Figure 9b and the evolutions for 10 °C and 25 °C can be seen in Supplementary Materials Figure S9. Regardless of the temperature, the introduction of GnP led to a decrease in the permeability compared to the PBS matrix at the same water activity. Once again, the impermeable GnP acts as a barrier and the dioxygen molecules should diffuse across a more tortuous route.

The activation energies for dioxygen permeation $E_{p\left(O_{2}\right)}$ were calculated from the Arrhenius equations. All of the obtained correlation factor values (*R^2^*) of the Arrhenius plot were higher than 0.99 (Supplementary Materials Figure S10). The activation energy for dioxygen permeation $E_{p\left(O_{2}\right)}$ as a function of water activity for the neat PBS and PBS/GnP nanocomposites are presented in Figure 9c. Regardless of the system, the $E_{p\left(O_{2}\right)}$ values remained constant, $(E_{p\left(O_{2}\right)}=$ 54 ± 5 kJmol^−1^), across the whole range of activity. The same tendency was also observed by Auras et al., for the evolution of the $E_{p\left(O_{2}\right)}$ values for PET and PLA for the water activity range of 0 to 0.9 [43]. The obtained value of $E_{p\left(O_{2}\right)}$ for PBS was in the same range order as the data reported in the literature. Salehi et al., reported $E_{p\left(O_{2}\right)}\;$values of 27 and 44 kJmol^−1^ for PP and PLA, respectively [44] and Auras et al., reported $E_{p\left(O_{2}\right)}$ values of 24 and 30 kJ.mol^−1^ for PLA and PET, respectively [43]. All of these values were obtained at an anhydrous state for a temperature range from 0 to 55 °C for Salehi et al. [44] and between 5 and 40 °C for Auras et al. [43]. This difference observed for a $E_{p\left(O_{2}\right)}$ value of PLA may be explained by the difference in the polymer crystallinity and the processing conditions.

After the addition of GnP, as observed for the water permeation analyses, there was no significant modification of $E_{p\left(O_{2}\right)}$ in the tested range of water activity compared to those obtained for the neat matrix.

There was a difference between both molecules in the value of the activation energy. Values were largely greater for $E_{p\left(O_{2}\right)}$ than the values for $E_{p\left(H_{2}O\right)}$, which could be attributed, at least partially, to the greater kinetic diameter of the dioxygen molecules compared to the water molecules, and the difference in the interactions between each molecule and the polymer. From these results, it could be confirmed that the activation energy was dependent on the studied permeant molecule.

## 5. Conclusions

Nanocomposites were prepared by melt process from a biodegradable polymer, PBS, and low amount of GnP. The influence of the coupled temperature and humidity on the transport properties of the neat PBS and the nanocomposites were investigated. The SEM analysis showed a good dispersion and a preferential orientation of GnP in the PBS matrix. Major conclusions from the water sorption analysis were the decrease in the solubility coefficient and the increase in the number of sorption sites per monomer unit in the amorphous phase (*N_i_/MCS*) when the temperature increased. Focusing on the diffusion of water through the studied films, at a constant temperature and for all samples, the diffusion coefficient was constant up to a water activity of 0.6, then decreased for higher water activity. At constant temperature and water activity, the diffusion coefficient decreased due to the addition of GnP in the PBS matrix, regardless of the studied temperature. This result was attributed to the increase in tortuosity induced by the presence of GnP and the presence of non-diffusive interfaces between PBS and GnP. On the other hand, the diffusion coefficients increased when the temperature increased on the neat PBS and PBS/GnP nanocomposites. This result was associated with the increase in molecular mobility of the amorphous phase of the matrix. The molar heat of the sorption and diffusion activation energy were calculated and revealed a strong correlation with the water activity and the clustering of water molecules in the PBS. In fact, for both the molar heat of sorption and the diffusion activation energy, for activity up to 0.7, the values were constant and decreased for higher activities. These results were attributed to the clustering of water molecules. The molar heat of sorption and the activation energy for diffusion were both not significantly modified when GnP was added to the PBS matrix.

Considering the water and dioxygen permeability, for both molecules, at a constant temperature and for all samples, the permeability coefficient increased when the activity increased. This result was attributed to the presence of sorbed water molecules that eased the permeability process. At a constant temperature and water activity, a gradual decrease in permeability was obtained when the amount of GnP increased due to the addition of tortuosity. At a fixed water activity and for all samples, the permeability for both molecules increased when the temperature increased on the neat PBS or PBS/GnP nanocomposites. These results might suggest that the permeability was, in this study, driven rather by the evolution of the diffusion of water molecules as the temperature increased. Once again, the activation energies of permeation were calculated and exhibited a strong difference between both molecules. The values of activation energy for permeation were lower for the water molecules (~15 ± 3 kJmol^−1^, at *a_w_* = 0.7) compared to the dioxygen molecules (~54 ± 5 kJmol^−1^, at *a_w_* = 0.7). For the water and dioxygen molecules, the activation energies remained almost constant across the whole range of water activity. Nevertheless, the addition of GnP did not lead to a significant change in the activation energy of permeation, and no significant modification of the activation energies for water or dioxygen permeability was observed as a function of the amount of GnP. Thus, this study confirms that the main reason for the improvement in the barrier properties to water by the addition of a low amount of GnP was the tortuosity effect, rather than a change in the transport mechanism.

## Figures and Tables

**Table 1:** The theoretical and real compositions determined from the thermogravimetric analysis (TGA) and sample code of the different films.

	Theoretical GnP Loading (wt.%)	Determined GnP Loading	
		wt.%	v.%
PBS	0	0.00 ± 0.00	0.00 ± 0.00
PBS/GnP0.1	0.1	0.06 ± 0.02	0.03 ± 0.01
PBS/GnP0.5	0.5	0.28 ± 0.12	0.16 ± 0.06
PBS/GnP1	1	0.66 ± 0.05	0.38 ± 0.03
PBS/GnP2	2	1.35 ± 0.02	0.78 ± 0.01

**Table 2:** The values of the glass transition temperature (*T_g_*) and crystallinity index (*X_c_*) of the neat PBS and corresponding composites (determined by DSC).

	PBS	PBS/GnP0.1	PBS/GnP0.5	PBS/GnP1	PBS/GnP2
*T_g_* (°C)	−35 ± 1	−36 ± 1	−36 ± 2	−36 ± 2	−37 ± 1
*X_c_* (%)	38 ± 1	38 ± 1	38 ± 1	38 ± 1	38 ± 1

**Table 3:** The values of the GAB parameters considering the sorption isotherm of the amorphous part of PBS and the corresponding nanocomposites at 10 °C, 25 °C, and 40 °C.

	M_m_ (×10^−3^)			C_g_			K			MRD (%)		
Temperature (°C)	10	25	40	10	25	40	10	25	40	10	25	40
Neat PBS	4.2	4.7	6.1	2.0	2.1	2.1	0.94	0.93	0.94	5.6	5.2	4.8
PBS/GnP0.1	4.2	4.6	5.9	2.0	2.1	2.2	0.94	0.93	0.91	5.7	5.1	3.5
PBS/GnP0.5	4.1	4.8	5.7	1.9	2.0	2.2	0.95	0.93	0.93	6.3	5.4	3.6
PBS/GnP1	4.1	4.8	5.7	2.1	2.1	2.4	0.93	0.94	0.91	5.6	5.5	2.7
PBS/GnP2	4.1	4.6	5.8	2.2	2.4	2.4	0.93	0.94	0.90	4.7	4.9	4.1

**Table 4:** The values of the heat of sorption Δ*H_s_*, activation energy for diffusion apparent *E_d_,* and activation energy for permeation $E_{p\left(H_{2}O\right)}$ calculated from Equation (15) for *a_w_* = 0.5 and determined from Equation (12).

	Sorption Experiment			Permeation Experiment
	Experimental		Calculated	Experimental
	∆*H_S_* (kJ mol^−1^)	*E_d_* (kJ mol^−1^)	$E_{p\left(H_{2}O\right)}$ (kJ mol^−1^)	$E_{p\left(H_{2}O\right)}$ (kJ mol^−1^)
Neat PBS	−36 ± 2	64 ± 4	28 ± 6	17 ± 2
PBS/GnP0.1	−36 ± 2	64 ± 4	28 ± 6	19 ± 2
PBS/GnP0.5	−36 ± 2	55 ± 3	19 ± 5	17 ± 2
PBS/GnP1	−35 ± 2	55 ± 3	20 ± 5	20 ± 2
PBS/GnP2	−36 ± 2	59 ± 4	22 ± 6	16 ± 2

## Data Availability

The data presented in this study are available in the Supplementary Materials.

## Supplementary Materials

The following supporting information can be downloaded at: https://www.mdpi.com/article/10.3390/membranes12070721/s1. Figure S1: Scanning electron micrographs of GnP particles, Figure S2: Mass gain (*X*-axis) and number of average number of water molecules sorbed in a single amorphous unit of polymer (Ni) (*Y*-axis) at a) 10 °C. (b) 25 °C and (c) 40 °C for neat PBS and corresponding nanocomposites, Figure S3: Solubility coefficient at (a) 10 °C. (b) 25 °C and (c) 40 °C for neat PBS and corresponding nanocomposites, Figure S4: Mean Cluster Size (MCS) at (a) 10 °C. (b) 25 °C and (c) 40 °C for neat PBS and corresponding nanocomposites, Figure S5: number of sorption site in the amorphous phase (Ni/MCS) at (a) 10 °C. (b) 25 °C and (c) 40 °C for neat PBS and corresponding nanocomposites, Figure S6: (a) Arrhenius plot used to calculate ∆Hs for neat PBS for some water activities and (b) Arrhenius plot used to calculate Ed for neat PBS for some water activities, Figure S7: Diffusion coefficient at (a) 10 °C. (b) 25 °C and (c) 40 °C for neat PBS and corresponding nanocomposites, Figure S8: Water permeability coefficient at (a) 10 °C. (b) 25 °C and (c) 40 °C for neat PBS and corresponding nanocomposites, Figure S9: Dioxygen permeability coefficient at (a) 10 °C. (b) 25 °C and (c) 40 °C for neat PBS and corresponding nanocomposites, Figure S10: (a) Arrhenius plot used to calculate (a) $E_{p\left(H_{2}O\right)}$ and (b) $E_{p\left(O_{2}\right)}$ for neat PBS for some water activities.
