# Supplementary material for: Effect of Temperature and Humidity on the Water and Dioxygen Transport Properties of Polybutylene Succinate/Graphene Nanoplatelets Nanocomposite Films
Source: Membranes (Basel). 2022 Jul 20;12(7):721. doi: 10.3390/membranes12070721 (PMC9316474; doi:10.3390/membranes12070721)
Supplement: Supplementary file 1 [file membranes-12-00721-s001.zip › membranes-1813510-supplementary.pdf]

Supplementary Materials

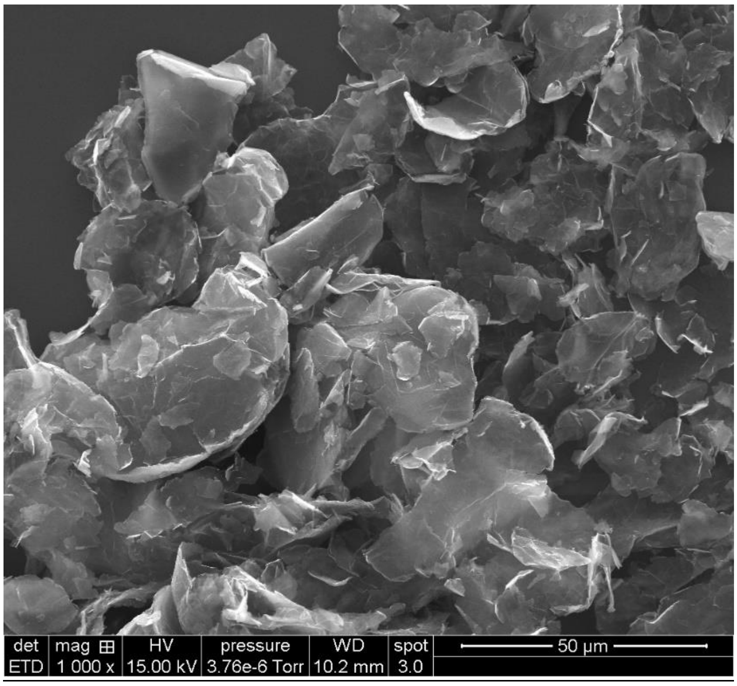

Figure S1: Scanning electron micrographs of GnP particles.

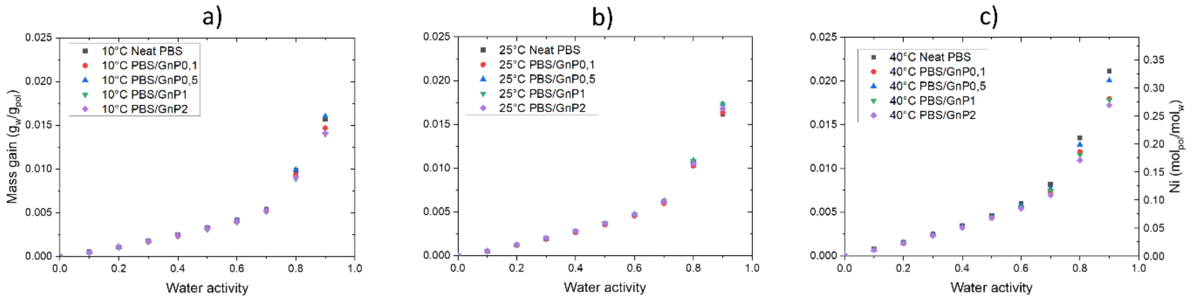

Figure S2: Mass gain (X-axis) and number of average number of water molecules sorbed in a single amorphous unit of polymer (*Ni*) (Y-axis) at a) 10 °C. b) 25 °C and c) 40 °C for neat PBS and corresponding nanocomposites.

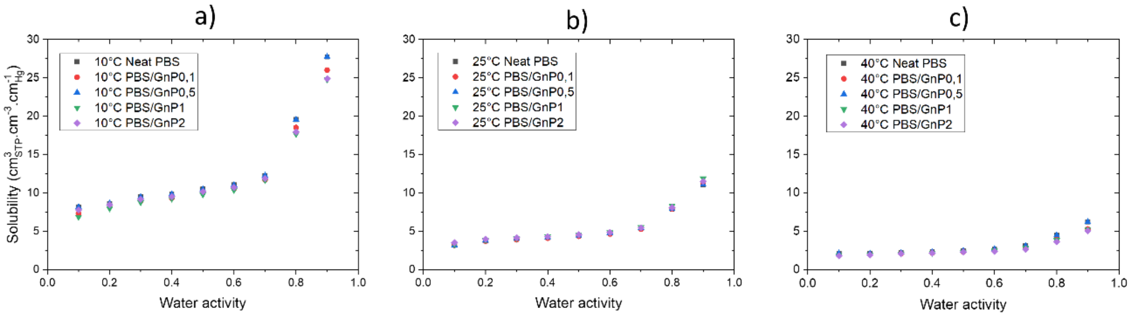

Figure S3: Solubility coefficient at a) 10 °C. b) 25 °C and c) 40 °C for neat PBS and corresponding nanocomposites.

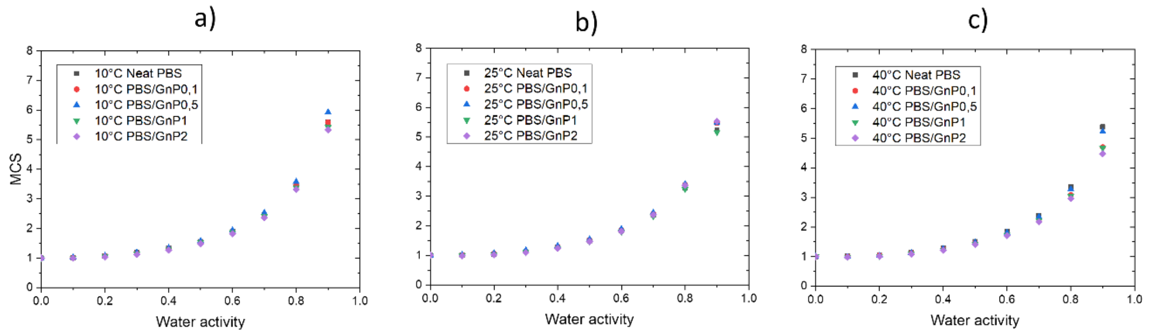

Figure S4: Mean Cluster Size (MCS) at a) 10 °C. b) 25 °C and c) 40 °C for neat PBS and corresponding nanocomposites.

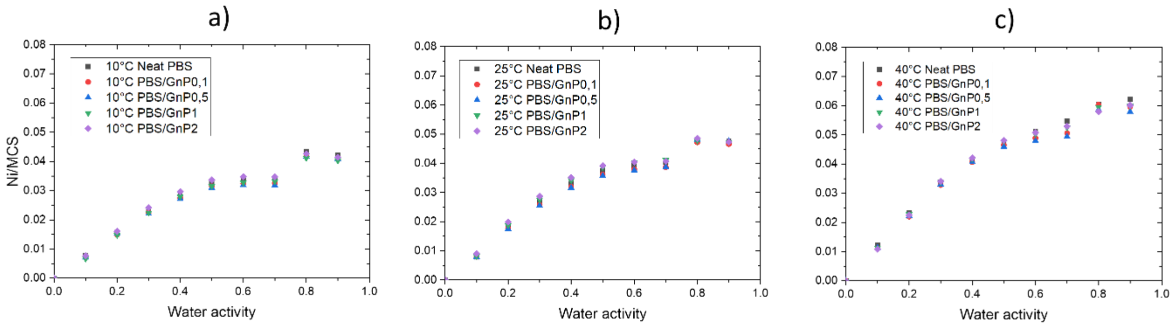

Figure S5: number of sorption site in the amorphous phase (*N_i/MCS*) at a) 10 °C. b) 25 °C and c) 40 °C for neat PBS and corresponding nanocomposites.

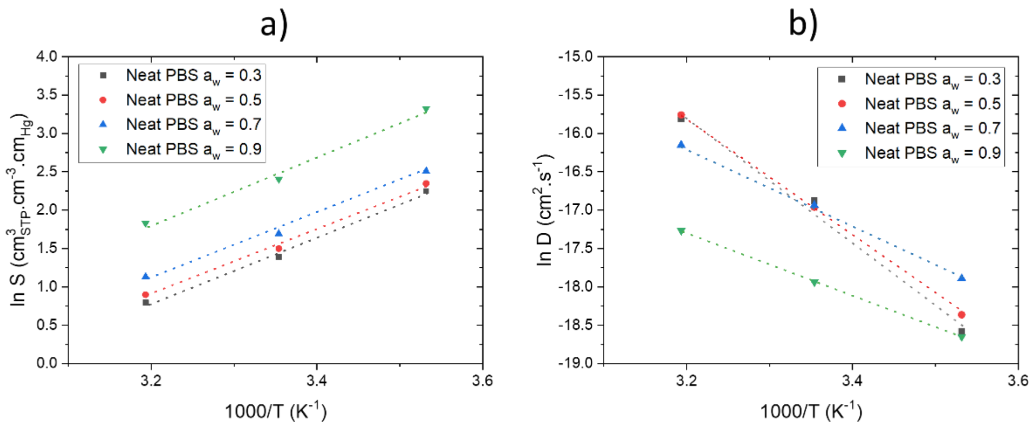

Figure S6: a) Arrhenius plot used to calculate ΔH_s for neat PBS for some water activities and b) Arrhenius plot used to calculate E_d for neat PBS for some water activities.

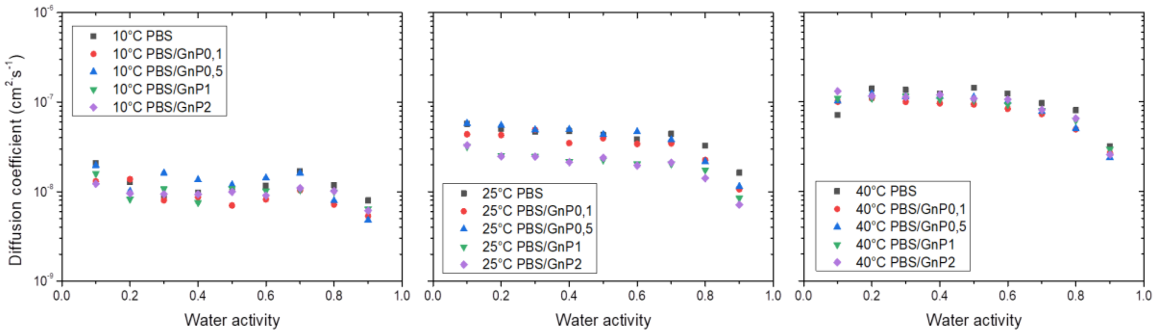

Figure S7: Diffusion coefficient at a) 10 °C. b) 25 °C and c) 40 °C for neat PBS and corresponding nanocomposites.

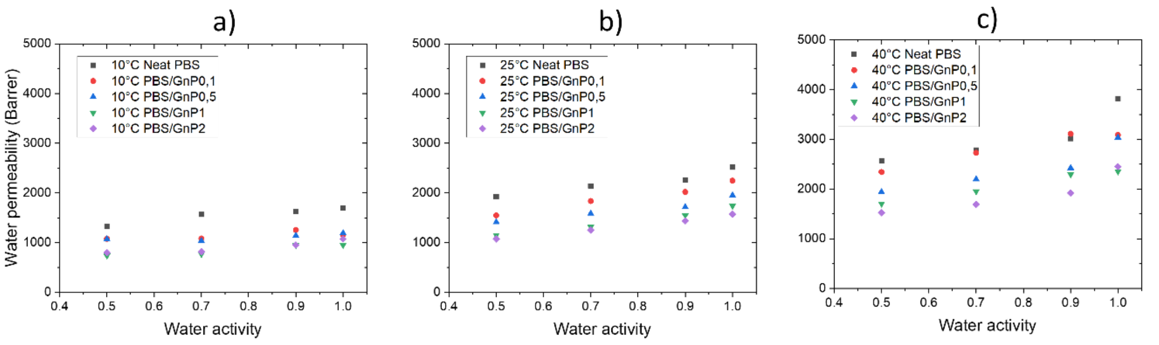

Figure S8: Water permeability coefficient at a) 10 °C. b) 25 °C and c) 40 °C for neat PBS and corresponding nanocomposites.

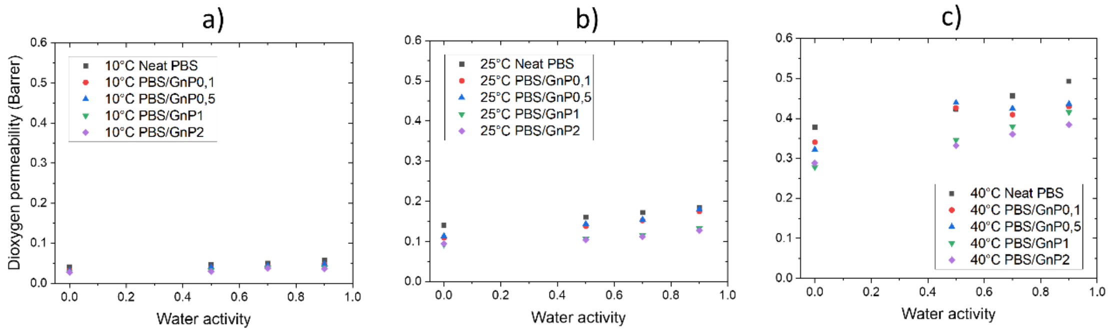

Figure S9: Dioxygen permeability coefficient at a) 10 °C. b) 25 °C and c) 40 °C for neat PBS and corresponding nanocomposites.

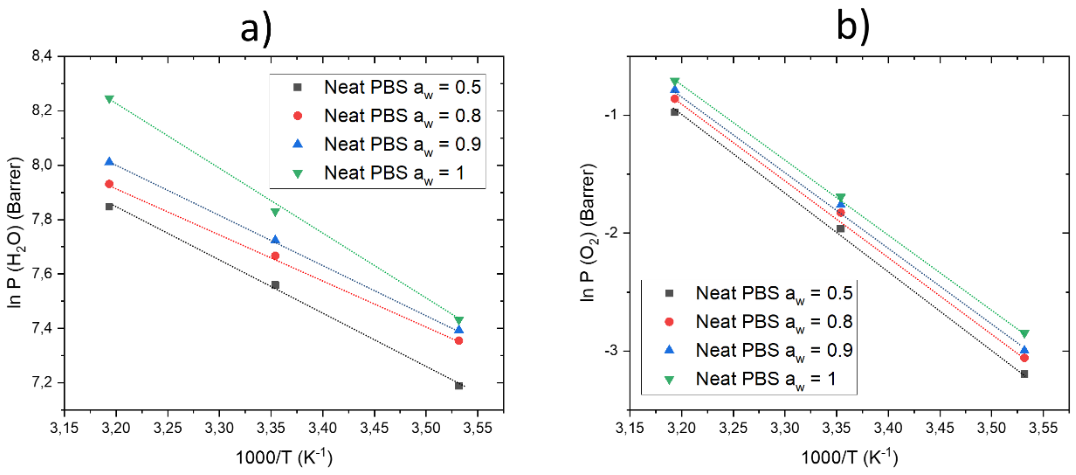

Figure S10: a) Arrhenius plot used to calculate a) E_p H_2O and b) E_p O_2 for neat PBS for some water activities.
